# The MMT of Elbow Flexion and the AFE Predict Impairment and Disability at 3 Weeks in Patients With Acute Stroke

**DOI:** 10.3389/fneur.2022.831800

**Published:** 2022-03-30

**Authors:** Shujiro Ueda, Hiroko Aoki, Yumiko Yasuda, Ayumi Nishiyama, Yusuke Hayashi, Kaoru Honaga, Akira Tanuma, Tomokazu Takakura, Akihiro Kurosu, Kozo Hatori, Akito Hayashi, Toshiyuki Fujiwara

**Affiliations:** ^1^Department of Rehabilitation Medicine, Juntendo University Urayasu Hospital, Chiba, Japan; ^2^Department of Rehabilitation Medicine, Juntendo University Graduate School of Medicine, Tokyo, Japan; ^3^Department of Physical Therapy, Juntendo University Faculty of Health Science, Tokyo, Japan

**Keywords:** rehabilitation, cerebrovascular disease, upper extremity, impairment, prediction

## Abstract

**Objective:**

This study aimed to investigate whether upper extremity motor function assessment within 72 h from stroke onset can predict the functional outcomes of the upper extremity.

**Design:**

This was a prospective, cohort study of patients with a first unilateral hemispheric stroke between May 2018 and March 2020. The motor arm item of the National Institutes of Health Stroke Scale, manual muscle testing of the elbow and forearm, and active finger extension scale were assessed within 72 h after stroke onset. The Fugl-Meyer assessment upper extremity motor score and action research arm test were assessed at discharge from the acute hospital. Multiple regression analysis was used to study predictors of upper extremity motor function at discharge from the acute hospital. The adjustment variables included age, sex, thumb localizing test, and visuospatial function.

**Results:**

Sixty acute stroke patients were recruited. The model with the highest coefficient of determination for the Fugl-Meyer assessment upper extremity motor score at discharge was the elbow flexion model (*R*^2^ = 0.76), followed by the active finger extension model (*R*^2^ = 0.69). For the action research arm test, the highest model was the active finger extension model (*R*^2^ = 0.64), followed by the elbow flexion model (*R*^2^ = 0.63).

**Conclusion:**

The manual muscle testing of elbow flexion and the active finger extension may be useful for predicting impairment and disability at 3 weeks in patients with acute stroke.

## Introduction

Upper extremity weakness is the most common impairment of stroke patients (1). Impairment of upper extremity motor function leads to activity limitations, participation restrictions, and reduced independence in daily life (2).

There are many assessments for upper extremity motor function such as the Fugl-Meyer assessment of upper extremity motor function (FMA-UE) (3), the National Institutes of Health Stroke Scale (NIHSS) motor arm item (4), manual muscle testing (MMT) (5, 6), and the active finger extension scale (AFE) (7). Recovery of hemiparesis was seen remarkably within 1 month after stroke onset (8). It is necessary to predict the functional outcome in the very early acute phase of stroke.

A meta-analysis for predicting upper extremity function in stroke reported that early upper extremity function is the most influential prognostic factor compared to other clinical factors and imaging factors (9). The FMA-UE is one of the good predictors of upper extremity motor function (10). The FMA-UE is assessed with the patient in the sitting position. It is, therefore, often difficult to assess the FMA-UE in the very early acute phase of stroke.

It has been reported that AFE, shoulder abduction, and grip strength within 72 h are useful for predicting upper extremity function (11, 12). It is very difficult to assess shoulder abduction and grip strength in patients with severe acute stroke because they have some difficulties in activities and seating posture. Simple tests, which can be assessed with the patient in the supine position, are needed to evaluate and predict upper extremity motor function in the very early acute phase of stroke. The AFE, NIHSS motor arm item, and some items of MMT can be performed in the supine position and can be used for clinical assessment of upper limb function in stroke. However, the connection and validity of the NIHSS motor arm, MMT, AFE, and clinical assessments (FMA-UE and ARAT) are unclear.

The purpose of this study was to investigate the early predictors of upper extremity motor function, which can be applied for very early acute stroke patients in bed in the resting supine position.

## Materials and Methods

### Participants

This prospective, cohort study included a convenience sample of patients with acute ischemic stroke and hemorrhagic stroke admitted to Juntendo University Urayasu Hospital. Data were collected from May 2018 to March 2020. Patients meeting the following criteria were included: older than 18 years of age; admitted with first unilateral hemispheric stroke; and written, informed consent was provided by the patient or family. Ischemic stroke and hemorrhagic stroke were defined according to the World Health Organization criteria. The type and localization of stroke were determined using computed tomography or magnetic resonance imaging. Exclusion criteria were inability to follow instructions, exacerbation or complications of stroke during hospitalization, limited upper extremity movement due to neurological conditions other than stroke (orthopedic disease, pain, or psychological problems), unable to evaluate within 72 h, and discharged or transferred <3 weeks after onset. Approval for this study was obtained from the institutional review board of Juntendo University Urayasu Hospital, and written, informed consent was obtained from all participants.

### Procedure

On the initial day of occupational therapy, the NIHSS motor arm item, MMT of the elbow and forearm, AFE, the thumb localizing test (13) for proprioception, and the visuospatial item of the Stroke Impairment Assessment Set (SIAS) (14) were measured. One week after onset, FMA-UE was measured. Three weeks after stroke onset, in addition to the above tests, FMA-UE and the action research arm test (ARAT) (15) were assessed.

### Assessment

#### National Institutes of Health Stroke Scale Motor Arm Item

The NIHSS is a tool used by healthcare providers to objectively quantify the impairment caused by a stroke (4). The NIHSS motor arm item examines the ability to hold the paralyzed upper extremity in space. The shoulder was flexed 45 degrees in the supine position. The score ranges from 4 (no movement) to 0 (holding for 10 s without drooping) (16).

#### Manual Muscle Testing

MMT is the most commonly used method for documenting impairments in muscle strength (5). Each muscle is tested manually and scored from 0 (no muscle contraction) to 5 (complete range of motion against gravity with full resistance). In this study, MMT of elbow flexion, elbow extension, forearm supination, and forearm pronation were performed. The method of MMT in the supine position is summarized in the Table 1. As for manual resistance, we carefully observed the patient's condition and performed it within the range that did not involve large fluctuations in blood pressure and pulse.

**Table 1:** The method of MMT in the supine position.

	**Grade**	**Evaluator**	**Patient**
MMT elbow flexion	0–2	Hold the patient's upper arm and wrist and assist the movement so that it is level with the floor	The shoulder joint should be in internal rotation Flex the elbow joint so that the forearm passes in front of the body.
	3–5		The shoulder joint in the middle position of internal and external rotation
MMT elbow extension	0–2	Hold the patient's upper arm and wrist and assist the movement so that it is level with the floor	The shoulder joint should be internally rotated Extend the elbow joint so that the forearm passes in front of the body
	3–5	Hold the patient's shoulder joint in 90 degrees of flexion	Extent the elbow joint toward the ceiling
MMT forearm pronation	0–5		The elbow joint flexion 90°
MMT forearm supination	0–5		The elbow joint flexion 90°

*MMT, Manual Muscle Testing*.

#### Active Finger Extension Scale

The AFE was developed by Smania et al. The patient was asked to actively extend all affected fingers except the first simultaneously, with the score ranging from 0 (absence of muscle contraction) to 5 (normal muscle power) (7).

#### Thumb Localizing Test

The thumb of the affected upper limb is held in space by the examiner. The patient is asked to grasp the thumb of the affected hand with the thumb and index finger of the unaffected upper limb. The deviation is scored from 3 (the patient is unable to find his thumb and does not climb up the affected arm to locate it) to 0 (the patient can locate the affected thumb accurately) (17).

#### Visuospatial Function

This is a screening test for unilateral spatial neglect included in SIAS. The examinee points to the center of a 50-cm tape measure, which is held by the examiner in front of the examinee. The deviation from the center is scored from 0 (more than 15 cm deviation from the central point) to 3 (<2 cm deviation from the central point) (14).

#### Fugl-Meyer Assessment Upper Extremity Motor Score

The Fugl-Meyer Assessment is a stroke-specific assessment scale based on the recovery process of hemiplegic stroke patients (3). The upper extremity motor score (33 items with a maximum score of 66 points) was used in this study. The FMA-UE is the most commonly used outcome measure of upper extremity impairment in prognostic studies (9).

#### Action Research arm Test

The ARAT consists of 19 items that are grouped into the following 4 subtests: grasp, grip, pinch, and gross movement. A maximum score of 57 was given based on the degree of completion and time for each action (15). ARAT is the most commonly used outcome measure of upper extremity function or functional movement in prognostic studies (9).

### Statistical Analysis

First, the correlations between age and measurements on the initial day of occupational therapy (NIHSS motor arm item, MMT of the elbow and forearm, AFE, thumb localizing test, and visuospatial item) were confirmed using Spearman's rank correlation coefficient. Then, the correlations among age, the measurements at 3 weeks (NIHSS motor arm, MMT of the elbow and forearm, AFE, thumb localizing test, and visuospatial item), FMA-UE at 3 weeks, and ARAT at 3 weeks were confirmed using Spearman's rank correlation coefficient. Finally, multiple regression analysis was performed to identify predictors of FMA-UE at 3 weeks using the stepwise method. Age, sex, thumb localizing test, and the visuospatial item were used as adjustment variables, and one of the NIHSS motor arm item, MMT of the elbow and forearm, or AFE was selected and entered as the independent variable. The FMA-UE at 3 weeks was assigned as the dependent variable. Then, the adjusted *R*^2^ for each model was compared. A similar test was conducted for ARAT at 3 weeks. Also, To compare our data with previous reports, we also split the sample into two groups based on FMA-UE 1 week > 10 and FMA-UE 1 week <11, given that the 70% rule was originally and subsequently shown to be upheld when FMA-UE 1 week > 10, but not when FMA-UE 1 week <11 (18, 19). All predictors were entered into the stepwise model, with *p* < 0.05 to enter and *p* > 0.10 to leave. We determined the goodness of fit adjusted *R*^2^, β coefficients, *p*, and analysis of variance (ANOVA; *F, p*) for each regression model. The significance level was set at 5%. All analyses were conducted using statistical software, SPSS 24.0 for Windows.

## Results

**Figure 1:**
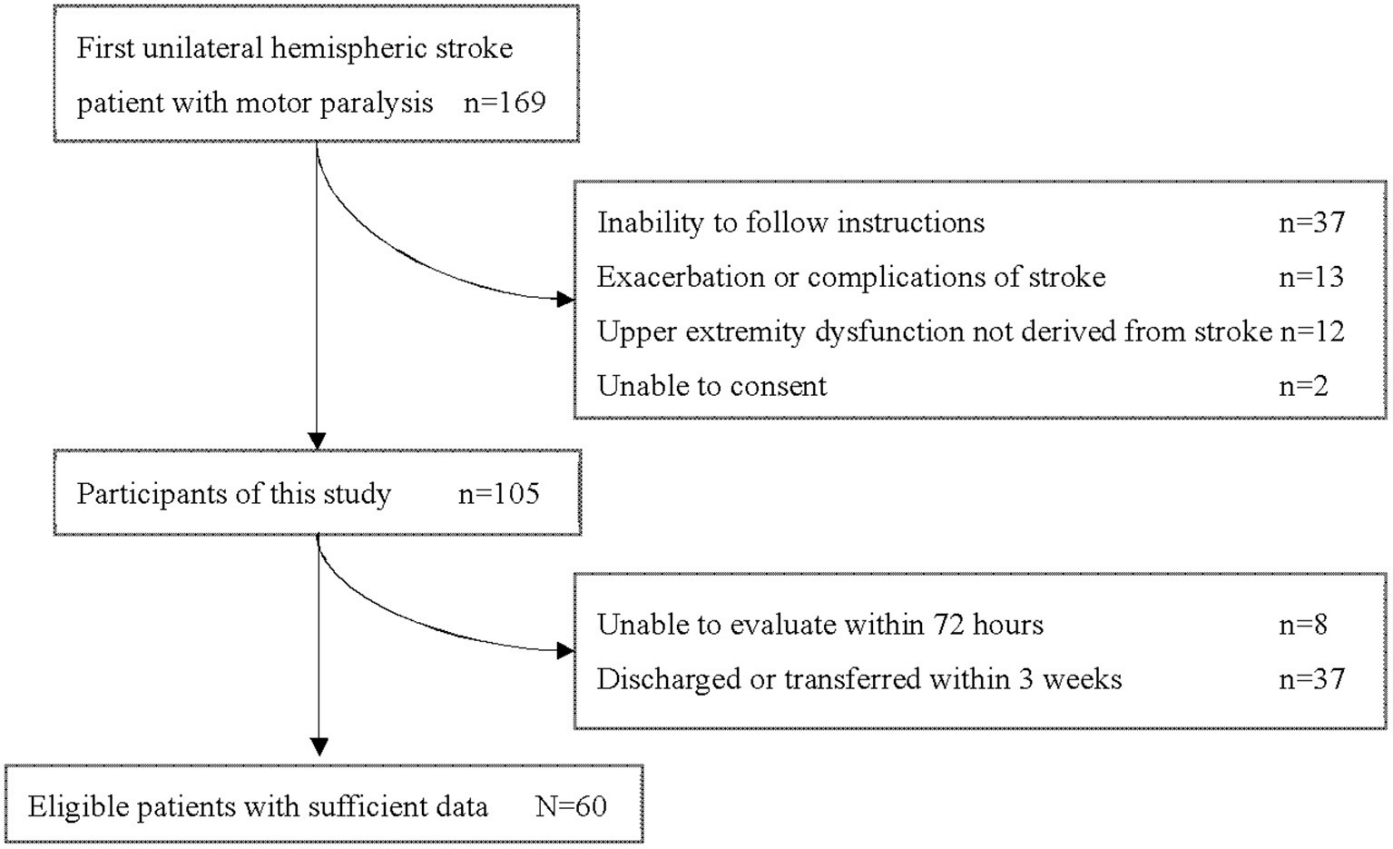
Flow chart of study participants.

During this study period, 169 patients aged 18 years or older were admitted to our hospital with a first unilateral hemispheric stroke and had motor paresis. Sixty patients (36 men, 24 women) with a median age of 68.0 (IQR 58.25–77.25) years who met the criteria were included in this study (Figure 1). Participants' characteristics are described in Table 2. There were 36 patients with cerebral infarction and 24 with cerebral hemorrhage. Of the 36 patients with cerebral infarction, one patient received both tissue plasminogen activator and endovascular therapy, eight patients received only tissue plasminogen activator. Craniotomy hematoma removal was performed for six of the 24 patients with cerebral hemorrhage. The median (IQR) number of days from stroke to start of occupational therapy was 1 (1, 2), and the median (IQR) number of days from stroke to the start of sitting was 2 (2–4). Thirty percent (18 of 60) of the patients were unable to receive FMA-UE within 72 h after stroke. The median (IQR) number of days of stay in our hospital was 29.5 (25–38.25). Fifty-six patients were transferred to inpatient rehabilitation facilities, two patients to home, and two patients to a chronic care facility.

**Table 2:** Participant Characteristics.

**Participant characteristics**		
Number of participants (male/female)	60 (36/24)
Age (years old, median, IQR)	68.0 (58.25–77.75)
Lesion type of stroke (infarction/hemorrhage)	36/24
Lesion side of stroke (left/right)	23/37
Days from stroke to start of OT (median days, IQR)	1(1–2)
Daysfrom stroke to start of sitting (range)	2 (2–4)
Length of hospital stay (medina days, IQR)	29.5 (25–38.25)
**Measurement**	Baseline	3 Weeks
NIHSS motor arm	3 (1–4)	2 (0–4)
MMT elbow flexion	2 (0–4)	3 (1–4.75)
Elbow extension	2 (0–4)	3 (1–5)
Forearm pronation	1.5 (0–4)	3 (1–5)
Forearm supination	1.5 (0–4)	3 (0–4.75)
AFE	1 (0–3)	1.5 (0–4)
Thumb localizing test	2 (1–3)	1 (0–2)
Visuospatial	2 (1.25–3)	3 (3–3)
FMA-UE	1week from onset 12.5 (4–52)	31.5 (8.25–59.5)
ARAT		4 (0–34)

*IQR, Interquartile range; OT, Occupational therapy; NIHSS, National Institutes of Health Stroke Scale; MMT, Manual Muscle Testing; AFE, Active Finger Extension; FMA-UE, Fugl-Meyer Assessment Upper Extremity; ARAT, Action Research Arm Test*.

**Figure 2:**
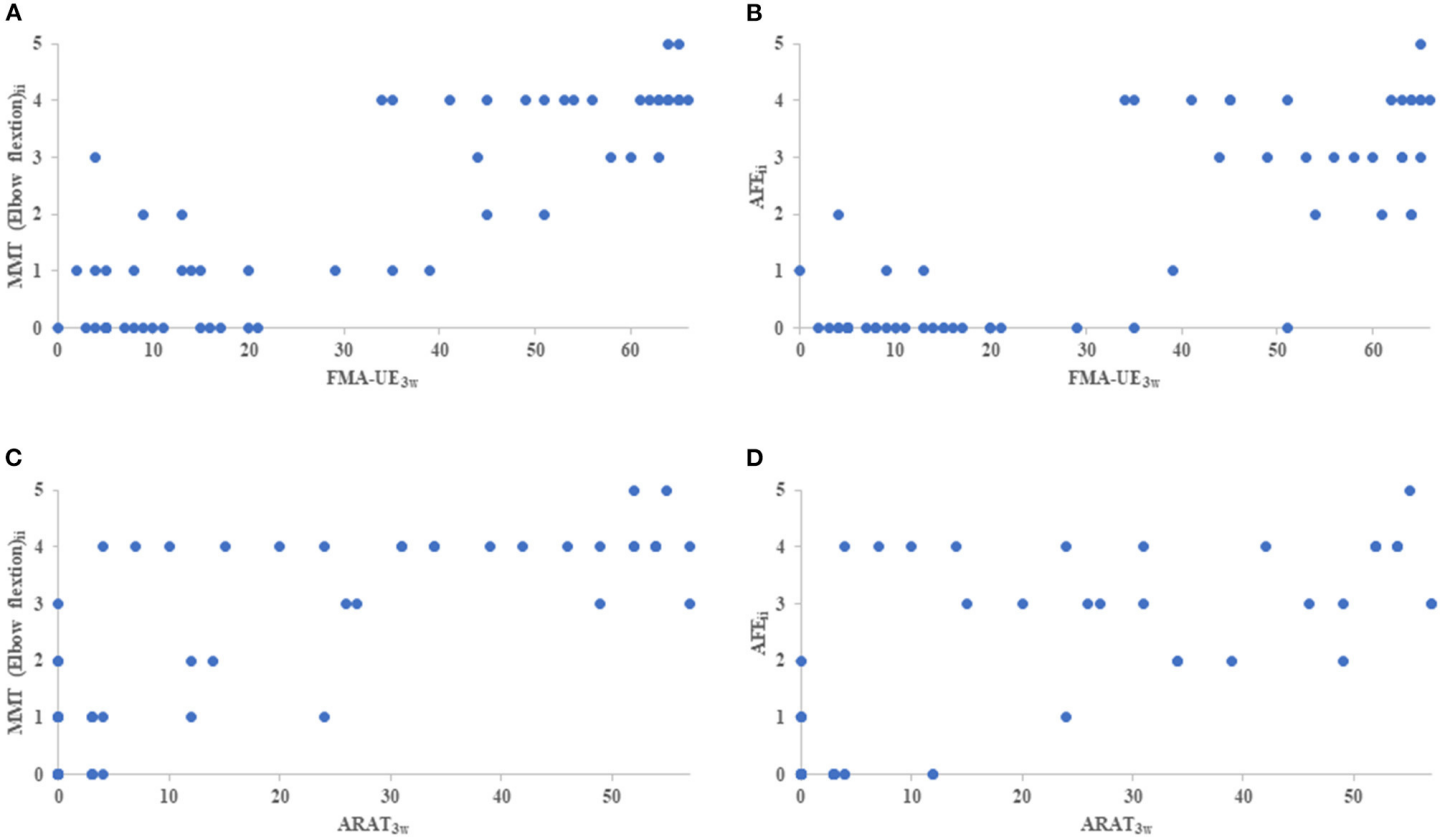
The correlation plot between initial impairment and clinical assessment of 3 weeks. **(A)** indicates MMT elbow flexion initial impairment (MMT elbow flexion_ii_) vs FMA-UE_3w_, **(B)** indicates AFE initial impairment (AFE_ii_) vs FMA-UE_3w_, **(C)** indicates MMT elbow flexion_ii_ vs ARAT, and **(D)** indicates AFE_ii_ vs ARAT. MMT, Manual Muscle Testing; AFE, Active Finger Extension; FMA-UE_3w_, Fugl-Meyer Assessment Upper Extremity at 3 weeks; ARAT_3w_, Action Research Arm Test at 3 weeks.

On the initial day of occupational therapy, the correlation coefficients of the NIHSS motor arm item, MMT of the elbow and forearm, and AFE showed strong correlations (*r* ≥ 0.88) with each other. The correlation coefficients of the NIHSS motor arm item, MMT of the elbow and forearm, and AFE at 3 weeks also showed strong correlations (*r* ≥ 0.87) with each other, and the correlation coefficients between these assessments and FMA-UE and ARAT were also very strong, with *r* > 0.90 (Table 3). The MMT elbow flexion initial impairment vs. FMA-UE at 3 weeks is shown in the correlation plot; ARAT is shown as well (Figure 2).

**Table 3:** Spearman's rank correlation coefficient between assessments at 3 weeks.

	**NIHSS motor arm**	**Elbow flexion**	**Elbow extension**	**Forearm pronation**	**Forearm supination**	**AFE**	**Thumb localizing test**	**Visuos patial**
NIHSS motor arm		−0.91^**^	−0.93^**^	−0.94^**^	−0.92^**^	−0.89^**^	0.52^**^	−0.38^**^
MMT elbow flexion	−0.91^**^		0.94^**^	0.94^**^	0.93^**^	0.92^**^	−0.57^**^	0.30^*^
MMT elbow extension	−0.93^**^	0.94^**^		0.94^**^	0.92^**^	0.87^**^	−0.58^**^	0.33^*^
MMT forearm pronation	−0.94^**^	0.94^**^	0.94^**^		0.95^**^	0.91^**^	−0.56^**^	0.33^*^
MMT forearm supination	−0.92^**^	0.93^**^	0.92^**^	0.95^**^		0.92^**^	−0.53^**^	0.30^*^
AFE	−0.89^**^	0.92^**^	0.87^**^	0.91^**^	0.92^**^		−0.51^**^	0.31^*^
Thumb localizing test	0.52^**^	−0.57^**^	−0.58^**^	−0.56^**^	−0.53^**^	−0.51^**^		−0.39^**^
Visuospatial	−0.38^**^	0.30^*^	0.33^*^	0.33^*^	0.30^*^	0.31^*^	−0.39^**^	
FMA-UE	−0.92^**^	0.93^**^	0.95^**^	0.94^**^	0.93^**^	0.92^**^	−0.59^**^	0.33^*^
ARAT	−0.94^**^	0.92	0.93^**^	0.93^**^	0.92^**^	0.91^**^	−0.53^**^	0.38^*^

** *P < 0.01*.

* *P < 0.05*.

*NIHSS, National Institutes of Health Stroke Scale; MMT, Manual Muscle Testing; AFE, Active Finger Extension*.

Multiple regression analysis to predict FMA-UE at 3 weeks showed that the model with the highest coefficient of determination was the elbow flexion model with an adjusted *R*^2^ = 0.76, followed by the AFE model with an adjusted *R*^2^ = 0.69. In the fitter group, the model with the highest coefficient of determination was MMT elbow flexion model with an adjusted *R*^2^ = 0.45, followed by MMT forearm pronation model with an adjusted *R*^2^ = 0.42. In the non-fitter group, there were no independent variables to be adopted in all models (Table 4). Multiple regression analysis was performed on the ARAT at 3 weeks, and the model with the highest coefficient of determination was the AFE model with an adjusted *R*^2^ = 0.64, followed by the elbow flexion model with an adjusted *R*^2^ = 0.63. In the fitter group, the model with the highest coefficient of determination was MMT elbow flexion model with an adjusted *R*^2^ = 0.37, followed by MMT forearm pronation model with an adjusted *R*^2^ = 0.35. In the non-fitter group, there were no independent variables to be adopted in all models (Table 5).

**Table 4:** Linear Regression Statistics for Predictors of FMA-UE at 3 weeks.

**Total**	**Model**	***Adjusted R* ^2^**	***F-*value**	***p***	**Predictor**	***B***	***p***
*N* = 60	NIHSS motor arm	0.67	121.6	<0.001	NIHSS motor arm	−0.823	<0.001
	MMT elbow flexion	0.76	183.8	<0.001	MMT elbow flexion	0.872	<0.001
	MMT elbow extension	0.62	97.1	<0.001	MMT elbow extension	0.791	<0.001
	MMT forearm pronation	0.67	120.9	<0.001	MMT forearm pronation	0.822	<0.001
	MMT forearm supination	0.65	108.1	<0.001	MMT forearm supination	0.807	<0.001
	AFE	0.69	66.4	<0.001	AFE	0.699	<0.001
					Thumb localizing test	−0.205	0.027
**Fitter**	**Model**	**Adjusted** ***R*^2^**	***F-*** **value**	***p***	**Predictor**	**β**	***P***
*N* = 33	NIHSS motor arm	0.35	18.3	<0.001	NIHSS motor arm	−0.610	<0.001
	MMT elbow flexion	0.45	27.4	<0.001	MMT elbow flexion	0.685	<0.001
	MMT elbow extension	0.31	15.0	<0.001	MMT elbow extension	0.571	0.001
	MMT forearm pronation	0.42	12.8	<0.001	MMT forearm pronation	0.689	<0.001
					Age	−0.291	0.044
	MMT forearm supination	0.27	12.9	<0.001	MMT forearm supination	0.542	<0.001
	AFE	0.25	11.4	<0.001	AFE	0.519	<0.001
**Non-fitter**	**Model**	***Adjusted R* ^2^**	***F*** **-value**	***p***	**Predictor**	**β**	***p***
*N* = 27	All				Nothing		

*FMA-UE, Fugl-Meyer Assessment upper extremity; NIHSS, National Institutes of Health Stroke Scale; MMT, Manual Muscle Testing; AFE, Active Finger Extension*.

**Table 5:** Linear regression statistics for predictors of ARAT at 3 weeks.

**Total**	**Model**	***Adjusted R* ^2^**	***F-*value**	***p***	**Predictor**	**β**	***p***
*N* = 60	NIHSS motor arm	0.59	87.2	<0.001	NIHSS motor arm	0.775	<0.001
	MMT elbow flexion	0.63	101.3	<0.001	MMT elbow flexion	0.797	<0.001
	MMT elbow extension	0.55	72.6	<0.001	MMT elbow extension	0.746	<0.001
	MMT forearm pronation	0.61	46.5	<0.001	MMT forearm pronation	0.802	<0.001
					Age	−0.175	0.039
	MMT forearm supination	0.57	77.6	<0.001	MMT forearm supination	0.757	<0.001
	AFE	0.64	36.4	<0.001	AFE	0.681	<0.001
					Age	−0.193	0.019
					Thumb localizing test	−0.220	0.026
**Fitter**	**Model**	**Adjusted** ***R*^2^**	***F-*** **value**	***p***	**Predictor**	**β**	***P***
*N* = 33	NIHSS motor arm	0.34	9.1	<0.001	NIHSS motor arm	−0.601	<0.001
					Age	−0.313	0.042
	MMT elbow flexion	0.37	10.6	<0.001	MMT elbow flexion	0.626	<0.001
					Age	−0.291	0.049
	MMT elbow extension	0.33	8.9	<0.001	MMT elbow extension	0.600	<0.001
					Age	−0.328	0.036
	MMT forearm pronation	0.35	9.6	<0.001	MMT forearm pronation	0.616	<0.001
					Age	−0.334	0.031
	MMT forearm supination	0.21	9.5	<0.001	MMT forearm supination	0.484	0.004
	AFE	0.31	8.2	<0.001	AFE	0.591	0.001
					Age	−0.356	0.028
**Non-fitter**	**Model**	**Adjusted** ***R*^2^**	***F-*** **value**	***p***	**Predictor**	**β**	***p***
*N* = 27	All				Nothing		

*ARAT, Action Research Arm Test; NIHSS, National Institutes of Health Stroke Scale; MMT, Manual Muscle Testing; AFE, Active Finger Extension*.

## Discussion

In this study, the median (IQR) number of days from stroke to starting sitting was 3 (3–5). Many patients are forced to stay on bed rest in the acute phase of stroke. Thirty percent (18 of 60) of the patients were unable to receive FMA-UE within 72 h after stroke. Although the study included patients with cerebral hemorrhage and postoperative stroke, many patients with cerebral infarction were also unable to receive FMA-UE. Previous studies that focus on patients within 72 h may have led to participant selection. Therefore, simple tests, which can be performed with the patient in the supine position, are needed to evaluate and predict upper extremity motor function in the very early acute phase of stroke. The NIHSS motor arm item, MMT of the elbow and forearm, and AFE could be assessed easily and safely in the supine position. Multiple regression analysis showed that MMT of elbow flexion and AFE were more predictive of upper extremity impairment and disability than the other assessments. The use of MMT of elbow flexion and AFE imposes minimal burden on the patient.

The decrease in adjusted *R*^2^ in the prediction for the fitter group may be partly the result of the halving of the number of cases. For the non-fitter group, we were not able to create a prediction model. This result supports a previous study (19), and the non-fitter group may include imperfections in the corticospinal tract. TMS assessment was not used in this study. We did not use TMS assessment in this study because we considered it difficult from the viewpoint of feasibility because of the inclusion of postoperative cases.

The AFE, shoulder abduction, and grip strength have been reported as simple prognostic evaluations (13, 14). Shoulder abduction and grip strength were excluded from the evaluation in the present study because of the difficulty of measuring them in the supine position. Since patients with unstable general conditions in the early stage of the disease, such as those in the postoperative period, were included in the study, we decided to exclude grip strength, which always requires maximal muscle exertion, from the viewpoint of clinical feasibility. The results of the present study showed that AFE and elbow flexion were more useful than the other assessments. It has been reported that AFE within 72 h is useful for predicting the prognosis of patients with upper extremity dysfunction (11, 12). The results of the present study were consistent with prior research. On the other hand, there have been few reports of the usefulness of elbow flexion alone. Malmut et al. (20) reported that Rapid bedside assessment of shoulder abduction, elbow flexion, and pinch grip using the arm subscore of the Motricity Index can predict upper limb recovery after stroke according to the ARAT. The MMT of elbow flexion may be useful in predicting upper extremity impairment and disability in the acute stroke rehabilitation setting.

In this study, detailed signs of spasticity are not known because assessment measures such as the Modified Ashworth Scale were not performed. Sixty participants in this study were able to be evaluated and measured with little effect of spasticity. The delay between acute neurological insult (trauma or stroke) and the appearance of spasticity (21) may be relevant. If the evaluation results are likely to be affected by spasticity, Repeated measures ought to be incorporated to examine reliability within a trial that includes participants with a hypertonic hand (22).

The present study showed that MMT of elbow flexion and AFE could be used to safely assess upper extremity function in patients with severe acute stroke. In this study, the outcome is 3 weeks after onset is that the average length of stay in acute care hospitals in Japan is around 3 weeks (median for both cerebral infarction 19.0 days and cerebral hemorrhage 27.0 days from All Japan Hospital Association FY2019 data) (23). Therefore, early prediction of the prognosis in the first 3 weeks after the onset of stroke is important when considering the subsequent rehabilitation plan and transfer to a rehabilitation hospital. Further work is needed to examine long-term outcomes by extending the prediction period. It is also desirable to identify simple and easy-to-understand assessments for patients with severe aphasia.

## Limitations

The present study has some limitations. The first is the shortness of the prediction period. Previous studies have often set a predictive period of 3–6 months after stroke (9). This study was limited to the acute phase of recovery. Second, the evaluation items were limited. Considering the patient's durability and comprehension, it is practically difficult to cover the entire upper extremity functional assessment. In addition, it is difficult to make distinctions between the NIHSS motor arm item and MMT of elbow and forearm because the measure and level of difficulty of the assessments are different. The third is the reliability of MMT in the supine position. Gravity assists the motion when the elbow flexion is more than 90 degrees, and gravity has little effect on MMT forearm pronation/supination test. It may be less reliable/valid than conventional MMT. There are AHA/ASA Guideline (24) recommendations for the use of MMT in stroke and its use as an outcome (25). However, as with shoulder abduction and AFE, intra- and inter-rater reliability of the test has not been demonstrated. Further work is needed. The fourth is the effect of multicollinearity in multivariate analysis and the small number of patients; considering the effect of multicollinearity in multivariate analysis, it is difficult to enter many variables in the evaluation of upper extremity function at the same time. The small number of cases in this study also limits the number of independent variables that can be assigned. The fifth is the bias of the participants. There was a difference in the number of participants on the lesion side because patients with symptoms of aphasia of left hemisphere stroke tended to be excluded. It was particularly difficult for them to understand the thumb localizing test and the visuospatial item. For this reason, the lesion side was not used for adjustment variables in the present multiple regression analysis. Considering these research limitations, it is difficult to generalize to all upper extremity dysfunctions.

## Conclusions

The NIHSS motor arm item, MMT of the elbow and forearm, and AFE can be assessed with the patient in the supine position in patients with severe acute stroke. The MMT of elbow flexion and AFE may be useful for predicting impairment and disability at 3 weeks in patients with acute stroke.

## Data Availability Statement

The original contributions presented in the study are included in the article/supplementary material, further inquiries can be directed to the corresponding author.

## Ethics Statement

The studies involving human participants were reviewed and approved by the Institutional Review Board of Juntendo University Urayasu Hospital. The patients/participants provided their written informed consent to participate in this study.

## Author Contributions

SU and TF developed the study, prepared and analyzed the data, interpreted the results, and wrote the manuscript. HA, YY, and AN recruited the participants, collected the data, collaborated in the data preparation, and reviewed the manuscript. YH worked on the data processing, data preparation, and reviewed the manuscript. KHo, AT, TT, AK, KHa, and AH developed the study, interpreted the results, and reviewed the manuscript. All authors contributed to the article and approved the submitted version.

## Conflict of Interest

The authors declare that the research was conducted in the absence of any commercial or financial relationships that could be construed as a potential conflict of interest.

## Publisher's Note

All claims expressed in this article are solely those of the authors and do not necessarily represent those of their affiliated organizations, or those of the publisher, the editors and the reviewers. Any product that may be evaluated in this article, or claim that may be made by its manufacturer, is not guaranteed or endorsed by the publisher.
